# The Oncologic Safety of Sentinel Lymph Node Biopsy in Patients with Node-Positive Breast Cancer with Complete Response to Neoadjuvant Chemotherapy: A Single-Center Experience

**DOI:** 10.1155/2023/4549033

**Published:** 2023-01-04

**Authors:** Ismail Can Tercan, Baha Zengel, Ozlem Ozdemir, Demet Cavdar, Funda Tasli, Zehra Hilal Adibelli, Murat Karatas, Cenk Simsek, Isabel Raika Durusoy, Adam Uslu

**Affiliations:** ^1^Department of General Surgery, Izmir Bozyaka Training and Research Hospital, Izmir, Turkey; ^2^Department of Medical Oncology, Izmir Bozyaka Health Practice and Research Center, Izmir, Turkey; ^3^Department of Pathology, Izmir Bozyaka Health Practice and Research Center, Izmir, Turkey; ^4^Department of Radiology, Izmir Bozyaka Health Practice and Research Center, Izmir, Turkey; ^5^Department of Public Health, Ege University, Medical Faculty, Izmir, Turkey

## Abstract

**Objective:**

To evaluate the efficiency and safety of sentinel lymph node biopsy (SLNB) in patients with breast cancer with complete response to neoadjuvant chemotherapy (NAC).

**Methods:**

Ninety-two consecutive (T1-4 and N1-2) patients with breast cancer who had pathologic and/or clinical and radiologic axillary lymph node involvement were included. All patients received NAC. Patients with a clinical and radiologic complete response in the axilla after NAC underwent SLNB. Pathologic complete response (ypCR) was defined as the absence of residual invasive and in situ cancer, and near-complete response (ypNCR) represented in situ and/or ≤ 1 mm residual tumor in the breast and/or presence of malignant cell clusters (≤0.2 mm) and/or micrometastases (≤2.0 mm) in the axillary lymph nodes (ALN) (ypTis/T1mi, ypN0i+/pN1mi).

**Results:**

The mean age of the 92 patients was 49.6 ± 10.3 years and the mean follow-up was 34.0 ± 17.8 months. With respect to breast tumors, 23 (25.0%) patients had complete and 14 (15.2%) had a near-complete response to NAC. Complete response in ALN was obtained in 39 (42.4%) patients and near-complete in six (6.5%) patients. The overall survival of the 33 patients who achieved ypCR and ypNCR was 100% and the remaining 59 patients with partial or no response to NAC was 83.1% at a mean follow-up of 34 months (*p*=0.063).

**Conclusions:**

In this study, no event developed in cases with ypCR and ypNCR in the breast and axilla. The persistence of the same results in long-termfollow-ups may enable the use of ypNCR as a positive prognostic marker in addition to ypCR.

## 1. Introduction

Neoadjuvant chemotherapy (NAC) was used in the 1970s to convert inoperable tumors into operable tumors and was later expanded to include early-stage breast cancer with an intent to perform breast-conserving surgery (BCS), especially for large tumors [1–3]. Taxanes combined with anthracycline-based chemotherapy considerably increased both the pathologic complete response (pCR) and the rate of BCS [4] and yielded favorable results in patients with triple-negative and HER2-positiveearly-stage breast cancer [5]. These data further extended the indication for NAC in the treatment of early-stage breast cancer to include tumors with high-risk biologic features. The standard approach for axilla in patients with early-stage breast cancer is sentinel lymph node biopsy (SLNB). Pathologic complete response rates for the axilla after NAC vary depending on the tumor subtype but range from 22% to 66.4% [6].

In this study, we aimed to retrospectively evaluate the oncologic safety of SLNB in patients with breast cancer whose axilla was pathologically and/or clinically and radiologically metastatic at the time of diagnosis and who had had a complete response after NAC.

## 2. Materials and Methods

Between 2013 and 2020, 92 consecutive (T1-4 and N1-2) patients with breast cancer with radiologically and/or pathologically proven axillary involvement, and those who underwent SLNB, following clinical and radiologic complete response after NAC, were included in the study. Patients with T0, N0, or in-situ tumors, inflammatory type and metastatic breast cancers, and those with no axillary response to NAC were excluded from the study. The patients were evaluated using breast ultrasonography (USG), mammography, and magnetic resonance imaging (MRI), and all of them underwent imaging-guidedtru-cut biopsy of the primary tumor and fine-needle aspiration biopsy (FNAB) and/or tru-cut biopsy of axillary lymph node(s). PET/CT was performed in the majority of patients for the assessment of metastasis. Of the patients who could have BCS, the tumor was marked with a metal clip under USG or mammography guidance.

In this study, NAC consisted of anthracyclines plus taxanes (AC or EC/T). All patients received an anthracycline-based regimen thrice weekly in the first four cycles (AC/T; doxorubicin 60 mg/m^2^, cyclophosphamide 600 mg/m^2^ or EC/T; epirubicin 90 mg/m^2^, cyclophosphamide 600 mg/m^2^) followed by either weekly paclitaxel (80 mg/m^2^) for 12 cycles or thrice-weekly docetaxel (80 mg/m^2^) for four cycles. In addition, patients with c-erbB2 positive luminal B tumors and c-erbB2-rich tumors received dual therapy with trastuzumab and pertuzumab. All patients were clinically and radiologically evaluated every two cycles of NAC, and unresponsive patients or those with progressive disease were excluded from the study and directed to surgical treatment.

## 3. Surgical Intervention

Breast-conserving or oncoplastic breast surgery was performed. Surgical specimens were sent for pathologic evaluation after complete tumor excision was proved using mammography. Our prerequisite for adequate and safe surgery was the removal of the lesion within negative margins on frozen sections (FS). Cavity revision was performed in patients with positive surgical margins. The remaining space after re-excision of the tumor was marked with metal clips for adjuvant radiotherapy planning. Mastectomy was performed in patients with extensive primary tumors and poor response to NAC.

The sentinel lymph node is identified with a dual method consisting of radiocolloid and isosulfan blue. Three or more sentinel lymph nodes (SLNs) were removed in each patient. They were immediately examined on FS or seldom left to the evaluation of paraffin blocks. Axillary lymph node dissection was performed in patients whose sentinel lymph nodes could not be identified and in those with positive SLNs.

All patients received 50 Gy adjuvant radiotherapy (RT) delivered in 25 fractions to the entire breast in those treated with BCS (BCS 10 Gy Boost 5 fractions) or to the chest wall for mastectomy and axillary (level I-II-III) and supraclavicular lymph node regions. Adjuvant trastuzumab treatment was administered to patients who were c-erbB2 positive for 1 year. Adjuvant hormone therapy was given to patients with hormone receptor-positive disease; tamoxifen was given to premenopausal patients and aromatase inhibitors to postmenopausal patients.

Pathologic response to NAC (yp) was classified as follows:

  Pathologic complete response (ypCR) was defined as the absence of residual invasive and in situ cancer on hematoxylin and eosin evaluations of the completely resected breast specimen and all sampled regional lymph nodes (i.e., ypT0 ypN0)
  Pathologic near-complete response (ypNCR) represented in situ and/or ≤ 1 mm residual tumor in the breast (ypTis/T1mi) and/or presence of pN0 (*i*+) malignant cell clusters (≤0.2 mm) and/or pN1mi micrometastases (∼200 cells, >0.2 mm, but ≤ 2.0 mm) in the axillary lymph nodes
  Partial response (ypPR): residual tumor burden in the breast > 1 mm and/or at least one metastasis in the axilla > 2.0 mm
  Unresponsive (ypNR): no response in the breast and axilla or progression of the disease

### 3.1. Statistical Analysis

Descriptive statistics are presented as frequencies and percentages for categorical data and means and standard deviations to summarize scale-type data. Kaplan–Meier survival analysis was conducted to estimate survival and to compare the differences in survival according to breast and lymph node responses to NAC. The patients' pre- and post-NAC pathologic parameters were compared using McNemar's test for categorical variables and pe- and post-NAC mean ER, PR, Ki67, and p53 values were compared using the *t*-test for dependent samples. Statistical significance was set at *p* < 0.05.

## 4. Results

The mean age of the 92 patients included in our study was 49.6 ± 10.3 years (minimum 30-maximum 77). At the time of diagnosis, 39.4% of the patients had a family history of breast cancer. Fifty-one (55.5%) patients were premenopausal and 41 (44.5%) were postmenopausal. The mean follow-up period was 34 ± 18 months. Axillary FNAB was not present in 21 patients (22.8%) who were referred from other centers. FNAB results were negative or nondiagnostic in 15 (16.3%) patients in our center. Of these 36 (30.1%) patients whose axillary metastasis could not be proved histopathologically, the diagnosis of axillary involvement was substantially based on physical examination findings and at least one of the accompanying axillary USG, Breast MRI, and PET/CT techniques. In the remaining 56 patients, the FNABs of 47 (51.1%) patients were reported positive, and nine (9.8%) were suspicious for metastasis. All patients received NAC and completed the treatment protocol. Thirty-three patients had c-erbB2 positivity. Of these, 25 received 12 months of adjuvant trastuzumab, and eight had dual therapy (trastuzumab + pertuzumab). Genetic counseling and testing were performed in 16 patients and no mutation was identified.

The clinical stages of the patients before and after NAC are shown in Table 1. Physical examination of the breast masses revealed a significant reduction in size after NAC (mean: 1.304 ± 1.109 cm vs. 0.071 ± 0.344 cm) (*p* < 0.001). The numbers of patients with clinically near-complete and complete responses after NAC were 16 (17.4%) and 18 (19.6%), respectively.

The pre- and post-NAC USG and MRI evaluation of the axilla and pathologic evaluation of the breast tumor are shown in Tables 2–4.

BCS was performed in 64 (69.6%) and mastectomy in 28 (30.4%) patients. SLNB was performed on 44 patients (47.8%). Axillary lymph node dissection (ALND) was performed on 36 (39.1%) patients with metastasis in SLNs in FS analysis and 12 (13.1%) patients whose SLN was not detected during surgery. SLNs were evaluated using intraoperative FS in 61 patients. The characteristics, pathology results, and complementary treatments of the patients whose SLNs were evaluated from paraffin blocks are shown in Table 5.

In terms of breast tumors, ypCR was obtained in 23 (25%) patients, ypNCR in 14 (15.2%) patients, and partial response in 52 (56.5%) patients after NAC. The response of axillary lymph nodes to NAC was ypCR in 39 (42.4%) patients, ypNCR in six (6.5%) patients, and ypPR in 30 (32.6%) patients. In patients receiving trastuzumab or dual therapy, ypCR and ypNCR rates of breast tumor were (50% vs. 50%) and (12.5% vs. 37.5%) (*p*=0.007), and for axillary lymph nodes they were (62.5% vs. 100%) and (6.3% vs. 0%) (*p*=0.007).

As surgical complications, the early-stage hematoma was detected in two patients and seroma was detected in one patient in the second year after surgery. These three patients were successfully treated with simple drainage. One patient presented with a soft-tissue infection in the remaining breast 4 years after surgery. The infection worsened during antibiotherapy and the patient underwent a mastectomy. A malignant tumor was observed in the contralateral breast of a patient in the second month after surgery. During the follow-up, locoregional recurrence did not develop in any of our patients.

Distant organ metastasis developed in nine patients. Liver metastases were detected in five patients, brain in two, bone in one, and bone plus liver metastasis in one patient. Two of these patients with brain metastases received surgical interventions; one underwent craniotomy and metastasectomy and the other had posterior fossa tumor surgery and external ventricular drainage.

**Figure 1:**
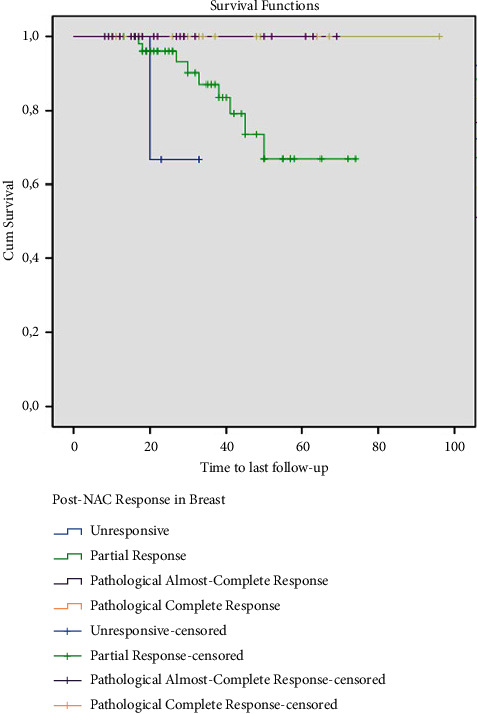
Survival of patients according to the response of their breast tumor to neoadjuvant chemotherapy (NAC) (months).

**Figure 2:**
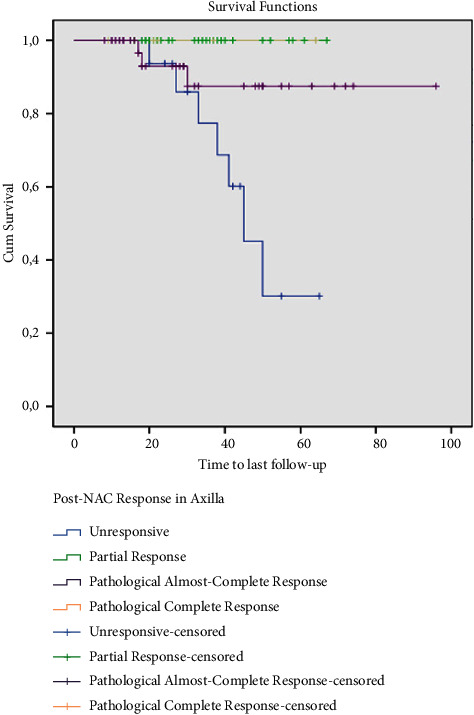
Survival of patients according to the response of their axillary lymph nodes to neoadjuvant chemotherapy (NAC) (months).

**Figure 3:**
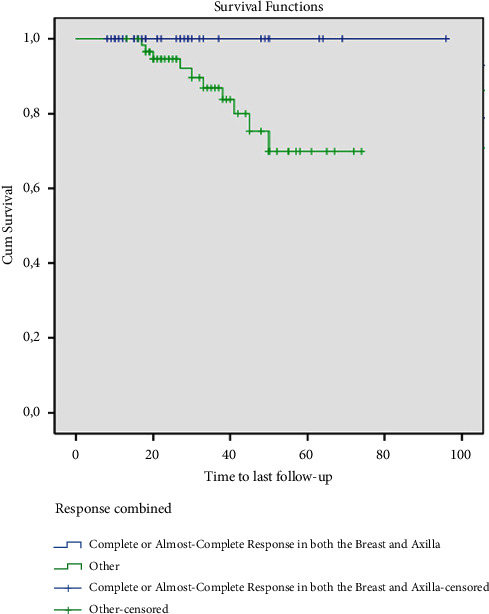
Survival analysis of patients with complete and near-complete response in both breast and axilla (months).

During the follow-up, 10 (10.9%) patients died. There was no mortality among the 37 patients who demonstrated ypCR and ypNCR in breast tumors after NAC. Survival at 74 months was 82.7% in patients with ypPR and at 33 months 66.7% in patients with ypNR (Figure 1). Also, estimated overall survival (OS) at 96 months was 92.3% and 100% in patients with ypCR and ypNCR in axillary lymph nodes after NAC, and at 65 months 58.8% in the unresponsive group (Figure 2). Finally, 33 patients with ypCR or ypNCR pathologic response to NAC both in the primary tumor and axillary lymph nodes and 59 patients with lesser response to NAC had an OS rate of 100% vs. 83.1% at a mean follow-up of 34 months (Figure 3).

## 5. Discussion

In this study, 92 consecutive female patients with clinical stage cT1-4 and N1-2 breast cancer underwent NAC to achieve clinical downstaging and subsequently perform as much BCS and SLNB as possible. Slightly different from the literature, we created a separate patient group under the name of near-complete response (ypNCR) to NAC based on the histopathologic evidence and very positive course in their clinical follow-ups. In our patients, there was 93.5% cT1-2 tumor and 6.5% cT3-4b tumor before NAC. As expected, following NAC, 23.9% of patients had cT0, 72.8% had cT1-2, and only one patient had a cT3 tumor. In the examination performed with imaging methods, a clinical complete response was obtained in the breast tumors of 19.6% of the patients (18/92) after NAC. These results were accompanied by a clinical complete response in the axillary lymph nodes of 98% of patients after NAC. With the assurance of these results, BCS was performed on 69.6% (64/92) and SLNB was performed on 57.6% (53/92) of the patients.

FNAB was performed on the axillary lymph nodes of 56 patients before NAC. Forty-seven of them (51.1%) had a definitive histopathologic diagnosis of metastases and nine patients (9.8%) had suspected metastases. Assuming that all of these 56 patients would otherwise undergo ALND, the administration of NAC reduced this number from 56 to 48 (*p*=0.3). Concerning breast tumors, 23 patients (25%) had a histopathologic complete response (ypCR) and 14 patients (15.2%) had a near-complete response (ypNCR) after surgery. In terms of axillary lymph nodes, there was ypCR in 39 (42.4%) patients, ypNCR in 6 (6.5%) patients, partial response in 30 (32.6%) patients, and no response in 17 (18.5%) patients. The estimated OS after NAC and surgery was 100% in patients with ypCR and ypNCR in breast tumors and 92.3% and 100%, respectively, in patients with ypCR and ypNCR in axillary lymph nodes.

Preliminary randomized studies investigating the efficacy of NAC containing doxorubicin and cyclophosphamide (AC) in patients with operable breast cancer achieved nearly the same 5-yeardisease-free and OS rates compared with the placebo group [7, 8]. In recent studies, the addition of taxane drugs to AC-based chemotherapy protocols has significantly improved relapse-free survival in these patients (hazard ratio (HR): 0.73; *p*=0.3). More importantly, the rate of BCS has increased significantly with the preoperative application of this combination (63% vs. 34%; *p* < 0.001) [4].

There are many studies in the literature showing the oncologic safety of SLNB alone after NAC in patients with breast cancer with clinical N1-N2 disease. For example, Wong et al. reported 11.3% recurrence among 211 women who underwent SLNB alone during a median follow-up of 36 months. The 5-year local recurrence rate was 5.7% for patients with cN0 disease and 4.1% for patients with the cN1-2 disease (*p*=0.55). For the cN0/ypN0 group and the cN1-2/ypN0 group, the 5-year axillary recurrence rate was 1.0% and 0% (*p*=0.44), respectively. This result accentuated the insignificance of clinical nodal status in patients who achieved ypN0 disease after SLNB [9]. Galimberti et al. reported acceptable locoregional recurrence rates for cN1-2/ypN0 patients. In their cohort of 147 node-positive patients, 48% of cN1-2 patients had negative SLNBs following NAC or NAC + endocrine therapy, and no axillary recurrence was observed during a median follow-up of 61 months [10]. Similarly, Martelli et al. evaluated the outcomes of cT2 cN0/1 patients using propensity score-weighted comparisons and reported no axillary recurrence with a 10-yeardisease-free survival (DFS) of 79% (95% CI: 68–92%) in patients who had SLNB-only and 69% (95% CI: 58–81%) in patients who underwent SLNB + axillary dissection (AD) [11]. Kahler-Ribeiro-Fontana et al. published their 10-yearfollow-up of 222 patients with cN1/N2 disease who became cN0 post-NAC. Of these 123 patients had negative SLNs and underwent SLNB alone. Despite a suboptimal number of SLNs retrieved (74% of the patients had ≤ 2 SLNs removed), only two (1.6%) of 123 ypN0 patients developed an axillary recurrence at a median follow-up of 9.2 years [12].

During a mean follow-up period of 34 ∓ 18 months, 10 of 92 (10.9%) patients deceased. There was no death among patients with ypCR and ypACR in breast tumors after NAC. However, the survival rates of patients who did not respond to NAC or had a partial response were 66.7% and 82.7%, respectively, and were statistically significantly lower than those with ypCR and ypACR (*p*=0.04).

Pathologic complete response (pCR) is the cornerstone for treatment success after NAC because NAC offers advantages such as higher BCS rates, and identifying subsets of patients with high pCR rates will allow surgeons to perform less invasive surgery. On the other hand, in the analysis of seven randomized studies, the hazard ratio for DFS was found as 0.446 in patients who developed pCR after NAC, and this ratio was observed as 0.523 in patients with in situ residues in breast tumors [13]. Although this second group refers to the ypNCR group in our study, we found no local or locoregional recurrence in this group. This is probably related to our short follow-up period. In a recent national study, it was reported that ALND could be safely avoided when certain conditions were met; for example, in meticulously selected cN-positive patients who underwent SLNB after NAC having breast and/or nodal pCR, cT1-2, or low volume residual nodal disease with luminal pathology, as long as axillary radiotherapy was provided [14]. Two prospective randomized clinical trials (NSABP-B51 and ALLIANCE) are still in progress, which hope to prove the oncologic safety of the axillary approach in patients who are node-positive receiving neoadjuvant systemic therapy, and the results will play an important role in determining the treatment in this patient group [15].

## 6. Conclusion

In this study, no event developed in cases with complete and almost complete histopathologic responses in breast tumors and axillary lymph nodes. The persistence of the same results in long-term follow-ups may enable the use of near-complete response as a positive prognostic marker in addition to complete response.

## Figures and Tables

**Table 1:** Clinical staging before and after neoadjuvant chemotherapy (NAC).

Clinical staging	Before NAC *n* (%)	After NAC *n* (%)
*cT*		
0	0 (0.0)	22 (23.9)
1	14 (15.2)	60 (65.2)
2	72 (78.3)	7 (7.6)
3	5 (5.4)	1 (1.1)
4b	1 (1.1)	0 (0.0)

*cN*		
0	0 (0.0)	90 (97.8)
1	82 (89.1)	2 (2.2)
2	10 (10.9)	0 (0.0)

*cM*		
0	92 (100.0)	92 (100.0)

*C stage*		
0	0 (0.0)	22 (23.9)
1A	1 (1.1)	60 (65.2)
2A	15 (16.3)	9 (9.8)
2B	62 (67.4)	1 (1.1)
3A	13 (14.1)	0 (0.0)
3B	1 (1.1)	0 (0.0)

**Table 2:** Evaluation of axilla with axillary USG before and after neoadjuvant chemotherapy (NAC) (*n*, row %).

Before NAC axillary USG	After NAC axillary USG			
	Negative *n* (%)	Regressed (partial response) *n* (%)	Positive	Total *n* (%)
Suspicious	8 (100.0)	0 (0.0)	—	8 (100)
Positive	58 (73.4)	21 (26.6)	—	79 (100)
Total	66 (75.9)	21 (24.1)	—	87 (100)

**Table 3:** Evaluation of axilla with axillary MRI before and after neoadjuvant chemotherapy (NAC) (*n*, row %).

Before NAC axillary MRI	After NAC axillary MRI			
	Negative *n* (%)	Regressed (partial response) *n* (%)	Positive *n* (%)	Total *n* (%)
Negative *n* (%)	3 (60.0)	2 (40.0)	0 (0.0)	5 (100)
Suspicious *n* (%)	2 (50.0)	2 (50.0)	0 (0.0)	4 (100)
Positive *n* (%)	42 (57.5)	29 (39.7)	2 (2.7)	73 (100)
Total *n* (%)	47 (57.3)	33 (40.2)	2 (2.4)	82 (100)

**Table 4:** Pathologic evaluation of breast tumors before and after neoadjuvant chemotherapy (*n*, column %).

Pathologic comparison	Before NAC *n* (%)	After NAC *n* (%)	*p*
Tumor pathology	(via tru-cut bx. of the breast)		
No tumor	0 (0.0)	23 (25.0)	
DCIS	0 (0.0)	3 (3.3)	NA
Infiltrating ductal carcinoma (IDC)	87 (94.6)	60 (65.2)	
Infiltrating lobular carcinoma (ILC)	3 (3.3)	2 (2.2)	
Other	2 (2.2)	4 (4.3)	

ER			McNemar *p*=
Negative	22 (23.9)	12 (13.0)	
Positive	66 (71.7)	57(62.0)	
Unavailable	4 (4.3)	23 (25.0)	
Mean (%) ± SD	76.53 ± 21.85	80.57 ± 23.99	Mean *p*=0.034

PR			McNemar *p*=
Negative	25 (27.2)	20 (21.7)	
Positive	64 (69.6)	48 (52.2)	
Unavailable	3 (3.3)	24 (26.1)	
Mean (%) ± SD	63.05 ± 32.40	55.05 ± 31.87	Mean *p*=0.098

CerbB2			
Negative	63 (68.5)	51 (55.4)	McNemar *p*=
Positive	29 (31.5)	16 (17.4)	
Unavailable	0 (0)	25 (27.2)	

Ki67			
Mean (%) ± SD	25.75 ± 15.16	20.36 ± 13.68	0.024

P53			
Mean (%) ± SD	35.83 ± 38.27	37.50 ± 39.12	<0.001

Histologic grade			
1		5 (5.4)	
2		37 (40.2)	
3		16 (17.4)	

Nuclear grade			
1		3 (3.3)	
2		28 (30.4)	
3		25 (27.2)	

Lymphovascular invasion			
Absent		58 (63)	
Present		11 (12)	

**Table 5:** Pathologic evaluation of paraffin sections of sentinel lymph nodes and complementary treatment.

	*n* (%)	Axillary dissection (ALND)
	19 pts. (100)	(No. of pathological lymph nodes/ No. of lymph nodes removed)
Negative	10 (52.6)	
Positive		
Isolated tm. Cells	2 (10.5)	ALND not applied
1+	2 (10.5)	ALND not applied
2+	3 (15.8)	Underwent ALND: 3 (0/5, 0/22, 0/18)
3+	1 (5.3)	Underwent ALND: 1 (2/13)
5+	1 (5.3)	Underwent ALND: 1 (4/8)

## Data Availability

Data are available from the corresponding author, Baha Zengel, upon reasonable request.
